# Entanglement-assisted quantum communication with simple measurements

**DOI:** 10.1038/s41467-022-33922-5

**Published:** 2022-12-22

**Authors:** Amélie Piveteau, Jef Pauwels, Emil Håkansson, Sadiq Muhammad, Mohamed Bourennane, Armin Tavakoli

**Affiliations:** 1 Department of Physics, Stockholm University, S-10691 Stockholm, Sweden; 2 Laboratoire d’Information Quantique, CP 225, Université libre de Bruxelles (ULB), Av. F. D. Roosevelt 50, 1050 Bruxelles, Belgium; 3 Hitachi Energy Research, Forskargränd 7, 72219 Västerås, Sweden; 4 Institute for Quantum Optics and Quantum Information - IQOQI Vienna, Austrian Academy of Sciences, Boltzmanngasse 3, 1090 Vienna, Austria; 5 Atominstitut, Technische Universität Wien, Stadionallee 2, 1020 Vienna, Austria

**Keywords:** Quantum information, Single photons and quantum effects

## Abstract

Dense coding is the seminal example of how entanglement can boost qubit communication, from sending one bit to sending two bits. This is made possible by projecting separate particles onto a maximally entangled basis. We investigate more general communication tasks, in both theory and experiment, and show that simpler measurements enable strong and sometimes even optimal entanglement-assisted qubit communication protocols. Using only partial Bell state analysers for two qubits, we demonstrate quantum correlations that cannot be simulated with two bits of classical communication. Then, we show that there exists an established and operationally meaningful task for which product measurements are sufficient for the strongest possible quantum predictions based on a maximally entangled two-qubit state. Our results reveal that there are scenarios in which the power of entanglement in enhancing quantum communication can be harvested in simple and scalable optical experiments.

## Abstract (web summary)

Quantifying communication capabilities produced by sharing an entangled qubit pair is still a subject of debate. Here the authors show that there are communication tasks for which sharing an entangled pair gives higher power than sharing two classical bits, even when there is no entanglement in the measurements.

## Introduction

Entanglement and quantum communication are both paradigmatic resources for quantum information science and crucial for understanding the nonclassical nature of quantum theory. The former has been studied for decades in Bell-type experiments^1–4^, where communication between the parties is not allowed. The latter has, in more recent years, been extensively studied in prepare-and-measure experiments, where shared entanglement is absent^5–7^. It is therefore natural to investigate the most general scenario, featuring both entanglement and quantum communication.

**Fig. 1:**
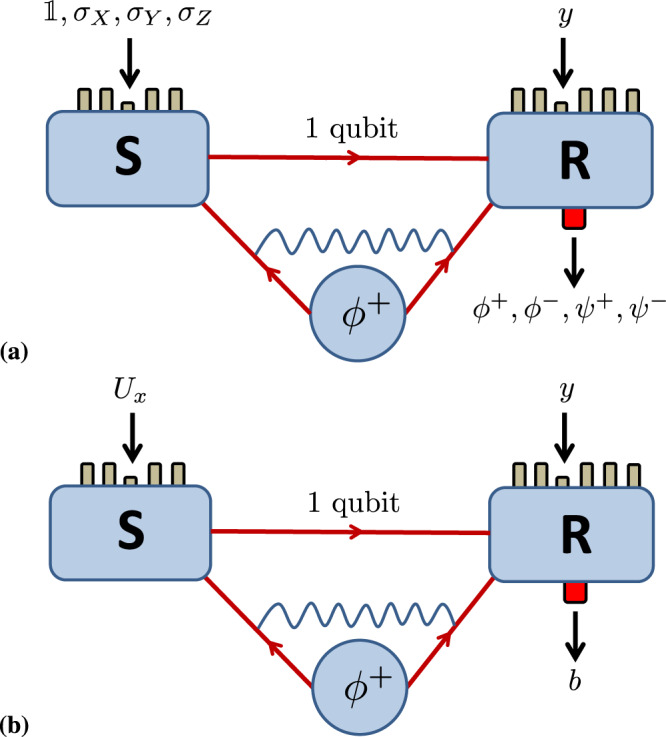
Entanglement-assisted quantum communication protocols. **a** *Dense coding scenario*. The sender (S) and receiver (R) share an EPR pair $\left|{\phi }^{+}\right\rangle$. The sender selects one of four Pauli unitaries $\{{\mathbb{1}},{\sigma }_{X},{\sigma }_{Y},{\sigma }_{Z}\}$, applies it to her qubit and relays it to the receiver who performs a measurement of both qubits in the basis of the four Bell states $\left|{\phi }^{\pm }\right\rangle=\frac{1}{\sqrt{2}}(\left|00\right\rangle \pm \left|11\right\rangle )$ and $\left|{\psi }^{\pm }\right\rangle=\frac{1}{\sqrt{2}}(\left|01\right\rangle \pm \left|10\right\rangle )$. From the outcome, the sender’s two bit input can be recovered. **b** *Generic sender-receiver scenario*. The parties again share an EPR pair and are allowed to communicate a qubit. The sender can now select between any number of arbitrary unitary operations *U*_*x*_ and the receiver can select between any number of arbitrary quantum measurements.

Dense coding is a striking illustration of the power of entanglement-assisted quantum communication^8^ (Fig. 1a). By sharing an Einstein-Podolsky-Rosen (EPR) pair, dense coding allows one to transmit two bits of classical information while sending only one qubit^8^. In contrast, a qubit alone can never carry more than one bit of information^9^. No entanglement-assisted protocol based on sending a qubit can transmit more than two bits^10^. Crucially, in addition to having an EPR pair, dense coding also requires the ability to jointly measure both shares in a basis of four maximally entangled two-qubit states; a so-called Bell basis measurement. For such a task, separable measurements do not offer any quantitative advantage over standard classical communication, regardless of the type of shared entangled state^11^.

However, in contrast to some other platforms for dense coding^12,13^, optical systems do not allow a straightforward implementation of a Bell basis measurement on separate photonic carriers. While optics is a particularly natural platform for quantum communication, such an implementation is impossible with linear optics^14,15^ unless one employs auxiliary degrees of freedom^16^. Nevertheless, two-qubit optical demonstrations of dense coding have been performed, for example by implementing deterministic partial Bell basis measurements that can in principle harvest at most ${\log }_{2}3$ bits^17^, or by encoding in continuous variables^18,19^, or by means of hyperentanglement which introduces additional photonic qubits^20–23^. Going beyond qubit systems, partial Bell basis measurements have recently been implemented on entanglement-assisted systems of dimension four to beat the two bit communication limit^24^. Nevertheless, deterministic implementations of sophisticated entangled measurements, in particular without auxiliary qubits, is difficult unless they are considerably restricted. Moreover, scaling a Bell basis measurement beyond the few lowest dimensions is an outstanding challenge.

Here we go beyond the dense coding task and consider more general communication tasks^25,26^ implemented with quantum messages assisted by entanglement. In such scenarios, when both entanglement and quantum communication are available, little is known about the predictions of quantum theory^27,28^. Here, we focus on the most elementary resources for such settings, namely a qubit message and a shared EPR state. We find that there exists correlation scenarios in which simple measurements can give rise to quantum correlations that cannot be simulated with two bits of classical communication, i.e., they cannot be reproduced with an ideal dense coding protocol. To this end, we first introduce a correlation task for which a standard partial Bell state analyser^29,30^ creates quantum correlations, that cannot be simulated with two bits of communication. Then, we go further and consider a well-established communication task, known as a Random Access Code, and show that product measurements are sufficient not only to elude classical models based on two bits of communication, but even to achieve the strongest predictions allowed by quantum theory for a two-qubit system. Thus, there exists natural communication tasks that can be implemented optimally by means of a quantum channel assisted by entanglement without the need for interference between the two photonic carriers in the measurement apparatus.

## Results

Consider a generic communication task in which the sender selects a classical input *x* and encodes it into a message that is sent to the receiver. The receiver selects a question, labelled *y*, to which he produces an answer labelled *b* (Fig. 1b). After many runs, they obtain probabilities *p*(*b*∣*x*, *y*). The parties pre-share the state $\left|{\phi }^{+}\right\rangle = \frac{1}{\sqrt{2}}\left[\left|00\right\rangle+\left|11\right\rangle \right]$ and the message consists of a single qubit, which is encoded via a local unitary *U*_*x*_ on the sender’s share. Once the receiver holds both shares, he performs the measurement {*E*_*b*∣*y*_}. The probabilities are given by the Born rule,

1 $$p(b|x , y) = {{{{{{{\rm{tr}}}}}}}}\left({E}_{b|y}({U}_{x}\otimes {\mathbb{1}}){\phi }^{+}({U}_{x}^{{{{\dagger}}} }\otimes {\mathbb{1}})\right).$$

Via dense coding, any *p*(*b*∣*x*, *y*), where *x* takes at most four values, can be generated in the experiment, regardless of the number of questions the receiver asks. Therefore, to find correlations that go beyond dense coding, one needs at least five values of *x*. We use the ability to beat classical communication models based on two bits as a basic benchmark for quantum protocols.

### Stochastic dense coding with Bell basis measurements

First, we show that there exists a natural information-theoretic task whose performance can be enhanced beyond what is possible with two bits. Consider a Random Access Code (RAC)^26^: the sender holds *x* = *x*_1_*x*_2_ ∈ {1, 2, 3, 4}^2^, and the receiver privately and uniformly selects *y* ∈ {1, 2} with the aim of recovering *x*_*y*_. This is a stochastic dense coding task, with average success rate

2 $${{{{{{{\mathcal{R}}}}}}}} = \frac{1}{32}\mathop{\sum}\limits_{x , y}p(b = {x}_{y}|x , y).$$

Via dense coding, the receiver can, e.g., always recover *x*_1_ but is then forced to guess the value of *x*_2_, yielding ${{{{{{{\mathcal{R}}}}}}}} = \frac{5}{8}$. In fact, no better two-bit strategy is possible (see “Methods”). Nevertheless, this bound can be exceeded using the same quantum resources. Let the receiver measure the bases

3 $$\left|{E}_{b|1}\right\rangle = {\mathbb{1}}\otimes {\sigma }_{X}^{{b}_{1}}{\sigma }_{Z}^{{b}_{2}}\left|{\phi }^{+}\right\rangle$$

4 $$\left|{E}_{b|2}\right\rangle = {\mathbb{1}}\otimes R\left|{E}_{b|1}\right\rangle , $$

where $R = \frac{1-i}{2\sqrt{2}}{\mathbb{1}}+\frac{1+i}{2\sqrt{2}}({\sigma }_{X}+{\sigma }_{Y}+{\sigma }_{Z})$ and *b* = *b*_1_*b*_2_ ∈ {0, 1}^2^. Given these measurements, the success rate is bounded by

5 $${{{{{{{\mathcal{R}}}}}}}} \le \frac{1}{32}\mathop{\sum}\limits_{x}\mathop{\max }\limits_{\{\left|{\psi }_{x}\right\rangle \}}\langle {\psi }_{x}|{E}_{{x}_{1}|1}+{E}_{{x}_{2}|2}|{\psi }_{x}\rangle \\ = \, \frac{1}{32}\mathop{\sum}\limits_{x}{\lambda }_{\max }({E}_{{x}_{1}|1}+{E}_{{x}_{2}|2}) = \frac{3}{4} , $$

where ${\lambda }_{\max }$ is the eigenvalue with the largest magnitude. This bound is reachable in our scenario because the eigenvector corresponding to ${\lambda }_{\max }$ for each *x* (the optimal two-qubit state) is maximally entangled, and hence realisable via a local unitary on $\left|{\phi }^{+}\right\rangle$. No better quantum protocol exists because the best protocol based on sending an unassisted four-dimensional quanta is known to achieve ${{{{{{{\mathcal{R}}}}}}}} = \frac{3}{4}$^31^, which must strictly bound the protocols of our interest from above. We refer to Supplementary Note 1 for a more detailed discussion of the Random Access Code, including the relation between its success probability and the classical capacity of (entanglement-assisted) communication channels.

However, the above advantage is based on performing a pair of (rotated) Bell basis measurements, i.e., measurements similar to that used in dense coding. These lack both simple implementation and scalability in dimension. Moreover, even though essential for dense coding, it may not be that such sophisticated measurements are necessary for more general entanglement-assisted quantum communications. We, therefore, proceed to investigate the usefulness of considerably more elementary measurements.

### Beyond two-bits models with a partial Bell state analyser

Consider a communication task with the minimal number of preparations needed to possibly beat two-bit protocols: the sender holds data *x* ∈ {1, …, 5} and the receiver selects questions *y* ∈ {1, …, 6}, each with a binary answer *b* ∈ {+1, −1}. Clearly, each question can only yield partial knowledge about *x*. We consider a simple figure of merit, ${{{{{{{\mathcal{S}}}}}}}}$, in which each question either has precisely one correct answer or no correct answer. We can rephrase this in terms of the answer “*b* = +1” either being awarded one point (if correct), being penalised by one point (if incorrect) or being ignored. Our figure of merit is

6 $${{{{{{{\mathcal{S}}}}}}}}\equiv \mathop{\sum }\limits_{x = 1}^{5}\mathop{\sum }\limits_{y = 1}^{6}{c}_{xy}p(b =+1|x , y) , $$

where the points awarded for each question are given by

7 $$c = \left(\begin{array}{cccccc}1&1&1&0&0&0\\ -1&0&0&1&0&0\\ -1&0&0&-1&1&0\\ 0&-1&0&-1&-1&1\\ 0&0&-1&-1&-1&-1\end{array}\right).$$

This figure of merit comes with favourable properties, but may be viewed as a proof-of-principle construction.

Using two bits of communication, the optimal score is ${{{{{{{{\mathcal{S}}}}}}}}}_{2}{{{{{{{\rm{bits}}}}}}}} = 5$ (see “Methods”). To saturate it, the two bits can be encoded as {*x* = 1, *x* = 2 ∨ 3, *x* = 4, *x* = 5}. Indeed, dense coding substantially improves on the best standard classical protocol, based on one bit of communication, (at best ${{{{{{{{\mathcal{S}}}}}}}}}_{{{{{{{{\rm{bit}}}}}}}}} = 3$). It also improves on the best protocol when one bit of communication is assisted by any amount of shared entanglement, specifically ${{{{{{{{\mathcal{S}}}}}}}}}_{{{{{{{{\rm{ent+bit}}}}}}}}}\;\approx\; 3.799$ (see “Methods”).

However, we can beat the two-bit limit: this time using only simple entangled measurements that only discriminate one of the four Bell states. Let the sender perform the following unitaries on her share of $\left|{\phi }^{+}\right\rangle$,

8 $$\begin{array}{c}{U}_{1}^{{{{{{{{\rm{S}}}}}}}}} = {\mathbb{1}} , \quad {U}_{2}^{{{{{{{{\rm{S}}}}}}}}} = \frac{-{\sigma }_{Z}\sqrt{3}-{\sigma }_{X}}{2} , \quad {U}_{3}^{{{{{{{{\rm{S}}}}}}}}} = \frac{{\sigma }_{X}\sqrt{3}-{\sigma }_{Z}}{2} , \\ {U}_{4}^{{{{{{{{\rm{S}}}}}}}}} = \frac{{\mathbb{1}}-i{\sigma }_{Y}\sqrt{3}}{2} , \quad {U}_{5}^{{{{{{{{\rm{S}}}}}}}}} = \frac{{\mathbb{1}}+i{\sigma }_{Y}\sqrt{3}}{2}.\end{array}$$

These are rotations in the *XZ*-plane of the Bloch sphere. Once the qubit is relayed, the receiver holds the state ${U}_{x}^{{{{{{{{\rm{S}}}}}}}}}\otimes {\mathbb{1}}\left|{\phi }^{+}\right\rangle$. The receiver performs a binary-outcome measurement $\{\left|{E}_{y}\right\rangle \left\langle {E}_{y}\right|, {\mathbb{1}}-\left|{E}_{y}\right\rangle \left\langle {E}_{y}\right|\}$, where the outcome *b* = +1 corresponds to a projection onto the state $\left|{E}_{y}\right\rangle = {U}_{y}^{{{{{{{{\rm{R}}}}}}}}}\otimes {\mathbb{1}}\left|{\phi }^{+}\right\rangle$ for some unitary ${U}_{y}^{{{{{{{{\rm{R}}}}}}}}}$. Such a measurement may be viewed as a locally rotated partial Bell state analyser; it attempts to discriminate the maximally entangled state $\left|{E}_{y}\right\rangle$ from its orthogonal complement. We choose the unitaries of the receiver as

9 $$\begin{array}{c}{U}_{1}^{{{{{{{{\rm{R}}}}}}}}} = {\mathbb{1}} , \quad {U}_{2}^{{{{{{{{\rm{R}}}}}}}}} = \frac{{\nu }_{+}{\mathbb{1}}+i{\nu }_{-}{\sigma }_{Y}}{2\sqrt{2}} , \quad {U}_{3}^{{{{{{{{\rm{R}}}}}}}}} = \frac{{\nu }_{+}{\mathbb{1}}-i{\nu }_{-}{\sigma }_{Y}}{2\sqrt{2}} , \\ {U}_{4}^{{{{{{{{\rm{R}}}}}}}}} = {U}_{2}^{{{{{{{{\rm{S}}}}}}}}} , \quad {U}_{5}^{{{{{{{{\rm{R}}}}}}}}} = {U}_{3}^{{{{{{{{\rm{S}}}}}}}}} , \quad {U}_{6}^{{{{{{{{\rm{R}}}}}}}}} = \frac{{\mathbb{1}}-i{\sigma }_{Y}}{\sqrt{2}}.\end{array}$$

where ${\nu }_{\pm } = \sqrt{3}\pm 1$. Once again, these are rotations in the *XZ*-plane. The figure of merit becomes

10 $${{{{{{{\mathcal{S}}}}}}}} 	= \, \mathop{\sum}\limits_{x , y}{c}_{xy}|\langle {E}_{y}|{U}_{x}^{{{{{{{{\rm{S}}}}}}}}} \otimes {\mathbb{1}}|{\phi }^{+} \rangle {|}^{2}\\ 	 = \, \frac{1}{2}{{{{{{{\rm{tr}}}}}}}}\left[{\left({U}_{y}^{{{{{{{{\rm{R}}}}}}}}}\right)}^{{{{\dagger}}} }{U}_{x}^{{{{{{{{\rm{S}}}}}}}}}\right] = 3+\frac{3\sqrt{3}}{2} \;\approx \;5.598 , $$

which considerably exceeds the two-bit limit. We note that the value (10) can be somewhat further increased. Using a numerical search, we find a protocol achieving ${{{{{{{\mathcal{S}}}}}}}}\approx 5.641$. However, this protocol is of lesser interest since it requires more complicated measurements. Our above protocol is also robust to unavoidable implementational imperfections. For instance, if the EPR pair is exposed to isotropic noise, so that the state becomes $v\left|{\phi }^{+}\right\rangle \left\langle {\phi }^{+}\right |+\frac{1-v}{4}{\mathbb{1}}$, for some visibility *v* ∈ [0, 1], the advantage over dense coding is maintained whenever $v\; > \, \frac{16}{12+3\sqrt{3}} \,\approx\, 93\%$.

**Fig. 2:**
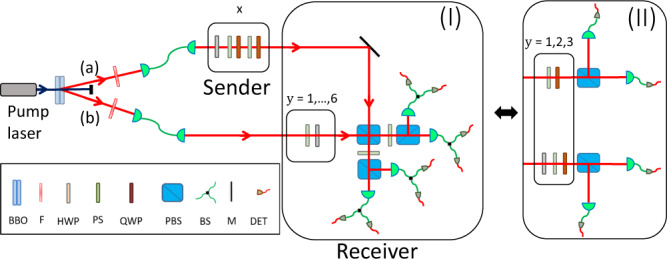
*Experimental setup*. Ultraviolet light centred at a wavelength of 390 nm is focused onto two 2 mm thick *β* barium borate (BBO) nonlinear crystals placed in cross-configuration to produce photon pairs emitted into two spatial modes (**a**) and (**b**) through the second order degenerate type-I SPDC process. The spatial, spectral, and temporal distinguishability between the down-converted photons is carefully removed by coupling to single mode fibre, narrow Filter (F) and quartz wedges respectively and prepare $\left|{\phi }^{+}\right\rangle$. The unitaries of the sender and receiver are implemented using combination of half wave plates (HWP), quarter wave plates (QWP) and phase shifters (PS). (I) The partial Bell state measurements are implemented through two-photon interference, using PBS and HWP plates set at 22.5^∘^. Beam splitters (BS) are introduced to estimate the projection probabilities before single photon detectors (actively quenched Si-avalanche photodiodes, DET). Outcome *b* = +1 corresponds to projection onto $\left|{\phi }^{+}\right\rangle$, and outcome *b* = −1 corresponds to the other Bell states $\left|{\psi }^{-}\right\rangle$, $\left|{\psi }^{+}\right\rangle$, and $\left|{\phi }^{-}\right\rangle$. In (II) partial Bell state measurement is replaced by product polarisation measurements and are performed by using HWPs, QWP and PBSs. Outcome *b* ≡ *b*_1_*b*_2_, with *b*_1_,*b*_2_ ∈ {+1, −1}^2^ corresponds to HH/VV or HV/VH detection when *b* = +1 or *b* = −1 respectively.

**Fig. 3:**
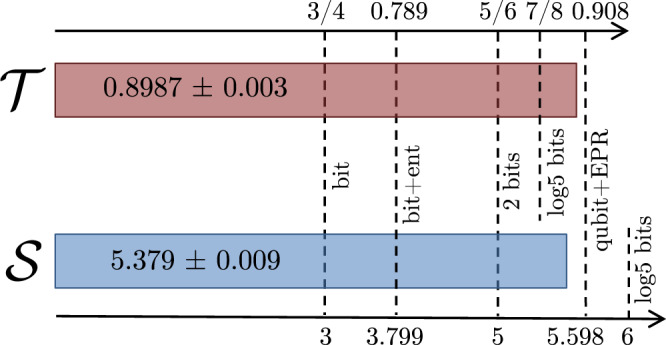
*Experimental results*. Illustration of the experimentally measured performance of the communication tasks and their comparison to the best conceivable protocols based on one bit of classical communication, one bit of classical communication assisted by unbounded entanglement, two bits of classical communication and five-valued classical communication. The two-bit bound is equal to the maximum attainable value using a dense coding protocol. Another dashed line represents the theoretical value of the targeted quantum protocol, based on a shared EPR pair and a communicated qubit.

Thanks to the simplicity of the measurements, the correlation advantage can be demonstrated using standard linear optics for polarisation qubits. Using a spontaneous parametric down-conversion (SPDC) process, we prepare two-photon polarisation entangled state $\left|\Psi \right\rangle = \frac{1}{\sqrt{2}}(\left|HH\right\rangle+\left|VV\right\rangle )$. The single-qubit unitaries are implemented using combinations of wave-plates and phase shifters while the partial Bell state measurement is implemented by interfering the two photons via a polarising beam splitter. The setup is illustrated in Fig. 2 and the specific settings are given in Supplementary Note 2. This leads us to the experimentally measured value of ${{{{{{{\mathcal{S}}}}}}}} = 5.379\pm 0.009$, which outperforms the dense coding limit by approximately 40 standard deviations (see “Methods”). Due to the sizeable violation and the large number of collected events, the *p*-value associated to our falsification of a two-bit model is vanishingly small (see “Methods”). The result and its relation to the various theoretical limits is illustrated in Fig. 3.

However, even though partial Bell state measurements offer sizeable advantages over two-bit protocols in relatively simple photonic experiments, they do not offer a clear path to scalability in terms of dimension or particle number. Also, from a conceptual standpoint, it is not self-evident that entanglement in the measurement must, in general, be indispensable for correlation advantages. To address both the practical and conceptual question, we investigate the possibility of using even the most elementary class of joint measurements, namely product measurements, for entanglement-assisted communication beyond two-bit protocols.

### Optimal Quantum Random Access Code with product measurements

Unfortunately, the previously discussed RAC seems to offer no advantage over two-bit classical models when the receiver is restricted to product measurements However, a variation of it, featuring a different number of inputs and settings, does reveal a distinct advantage. Let the sender hold data *x* = *x*_1_*x*_2_*x*_3_ ∈ {0, 1}^3^ and the receiver uniformly select *y* ∈ {1, 2, 3} with the aim of recovering *x*_*y*_. The average success rate is

11 $${{{{{{{\mathcal{T}}}}}}}} = \frac{1}{24}\mathop{\sum}\limits_{x , y}p(b = {x}_{y}|x , y).$$

A two-bit protocol achieves at best ${{{{{{{{\mathcal{T}}}}}}}}}_{{{{{{{{\rm{2bit}}}}}}}}} = \frac{5}{6}$ which can be shown considering all vertices of the classical polytope (see “Methods”). It is saturated by the sender relaying both the majority bit in *x* and the *x*_3_ to the receiver. Now consider that the sender performs the unitaries

12 $${U}_{x} = {(-1)}^{{x}_{1}}\left(\begin{array}{cc}-{\alpha }_{{x}_{1}}{\mu }_{{x}_{2}{x}_{3}}&{(-1)}^{{x}_{2}+{x}_{3}}{\alpha }_{{\bar{x}}_{1}}{\mu }_{{x}_{2}{x}_{3}}\\ {(-1)}^{{x}_{2}+{x}_{3}}\sqrt{2}{\alpha }_{{\bar{x}}_{1}}&\sqrt{2}{\alpha }_{{x}_{1}} , \end{array}\right)$$

where ${\mu }_{{x}_{2}{x}_{3}} = {(-1)}^{{x}_{2}}+i{(-1)}^{{x}_{3}}$, ${\alpha }_{s} = \frac{1}{2}\sqrt{1+{(-1)}^{s}\sqrt{2/3}}$ and the bar-sign denotes bit-flip. The receiver measures three product observables

13 $${E}_{1} = {\sigma }_{Z}\otimes {\sigma }_{Z}$$

14 $${E}_{2} = \frac{1}{2}{\sigma }_{Y}\otimes \left(\sqrt{3}{\sigma }_{Y}+{\sigma }_{Z}\right)$$

15 $${E}_{3} = \frac{1}{2}{\sigma }_{X}\otimes \left(\sqrt{3}{\sigma }_{Y}-{\sigma }_{Z}\right) , $$

where *E*_*y*_ ≡ *E*_1∣*y*_ − *E*_2∣*y*_. This leads to ${{{{{{{\mathcal{T}}}}}}}} = \frac{1}{2}+\frac{1}{\sqrt{6}} \; > \; {{{{{{{{\mathcal{T}}}}}}}}}_{{{{{{{{\rm{2bit}}}}}}}}}$. In fact, this outperforms even protocols based on sending five classical symbols. Interestingly, no better quantum protocol is possible, even if based on general entangled measurements. This follows from the fact that the best protocol based on four-dimensional quanta is also known to achieve ${{{{{{{\mathcal{T}}}}}}}} = \frac{1}{2}+\frac{1}{\sqrt{6}}$^32^. Adapting the optical setup (see Fig. 2), we have demonstrated also this correlation advantage using the same source and the same measuring time as in the previous experiment. The specific settings are given in Supplementary Note 4. We observe ${{{{{{{\mathcal{T}}}}}}}} = 0.8987\pm 0.003$, which beats the two-bit limit with over 20 standard deviations (see “Methods”). The results are illustrated in Fig. 3.

## Discussion

The finding, that simple measurements are sufficient for creating quantum correlations that cannot be modelled with two bits of classical communication, and sometimes even constitute an optimal protocol for natural quantum resources, is based on departing from the study of the dense coding task in favour of more general quantum communication tasks. Conceptually, it motivates a research effort into general entanglement-assisted correlations^27,28^ based on product measurements for the receiver. A natural question is to determine when and why product measurements are useful for entanglement-assisted quantum communication. It is paired with crucial practical advantages since such protocols circumvents the need for implementing highly demanding entangled measurements in favour of quantum devices that require only single-system measurements. This may make possible both multi-particle and high-dimensional protocols for entanglement-assisted quantum communication that are realistically implementable. It may also offer a viable practical path to otherwise demanding foundational experiments based on these natural quantum resources.

## Methods

### Correlation bounds

When communication is classical and no entanglement is present, *p*(*b*∣*x*, *y*) can be geometrically represented as a polytope whose vertices correspond to deterministic encoding and decoding schemes^5^. Consequently, the optimal performance of any linear figure of merit, e.g., that in Eq. (6), is necessarily attained at a vertex of this polytope. One can thus check the value of the figure of merit at all vertices and select the largest value. However, when (potentially unbounded) entanglement is added, this picture breaks down. Instead, upper bounds on ${{{{{{{\mathcal{S}}}}}}}}$ and ${{{{{{{\mathcal{T}}}}}}}}$ can be determined using the hierarchy of semidefinite programming relaxations developed in ref. 27, which uses the concept of informationally-restricted quantum correlations^33,34^. Using this method, and matching it with an explicit entanglement-based strategy with classical communication, we find ${{{{{{{{\mathcal{S}}}}}}}}}_{{{{{{{{\rm{ent+bit}}}}}}}}}\;\approx\; 3.799$ and ${{{{{{{{\mathcal{T}}}}}}}}}_{{{{{{{{\rm{ent+bit}}}}}}}}}\;\approx\; 0.789$.

The optimality of our protocols for the two RACs, corresponding to ${{{{{{{\mathcal{R}}}}}}}}$ and ${{{{{{{\mathcal{T}}}}}}}}$, respectively, can be shown as follows. For both results, we use that the set of correlations attainable with an EPR pair and a qubit message is a subset of the set of correlations attainable in scenarios in which the sender and receiver only share classical randomness and communicate a four-dimensional quantum system. In these standard scenarios, it is known that ${{{{{{{\mathcal{R}}}}}}}}\le \frac{3}{4}$^31^ and that ${{{{{{{\mathcal{T}}}}}}}}\le \frac{1}{2}+\frac{1}{\sqrt{6}}$^32^ for general protocols. As our protocols saturate these bounds, optimality follows.

### Experimental errors

To reduce the multi-photons pairs emission we worked at a low rate (≈2500 two-photon coincidences per sec, ca. 13% of the singles rate) and increased the measurement time to reduce statistical errors. We benchmark the state preparation by measuring an average visibility of 0.992 ± 0.001 in the diagonal polarisation basis. Similarly, we benchmark the two-photon interference by a two-fold Hong–Ou–Mandel dip visibility of 0.961 ± 0.002 (see Supplementary Note 7). For each setting *x* and *y*, we collect on average 18 million events during a measurement time of two hours. The probabilities *p*(*b*∣*x*, *y*) are estimated from the relative frequencies (see Supplementary Notes 3 and 5). The impact of systematic errors was estimated using Monte Carlo simulation. These were reduced by using computerised high precision mounts (see details in Supplementary Note 6). The experiment is performed using the fair sampling assumption.

### Statistical significance

To express the statistical significance of our experimental results, we follow an approach similar to^35^ introduced by^36^, to which we refer for details. Consider the random variable

16 $${\hat{{{{{{{{\mathcal{S}}}}}}}}}}_{i} = \mathop{\sum}\limits_{xy}{c}_{xy}\frac{\chi ({b}_{i} = 1 , {x}_{i} = x , {y}_{i} = y)}{p(x , y)} , $$

where *i* corresponds to the *i*th experimental run, *χ*(*e*) is the indicator function for the event *e*, i.e., *χ*(*e*) = 1 if the event is observed and *χ*(*e*) = 0 otherwise. For our experiment we simply chose *p*(*x*, *y*) = 1/(6 × 5) = 1/30. The random variable ${\hat{{{{{{{{\mathcal{S}}}}}}}}}}_{i}$ may depend on past events, *j* < *i*, but not on future events, *j* > *i*. We define $\hat{{{{{{{{\mathcal{S}}}}}}}}} = \frac{1}{N}\mathop{\sum }\nolimits_{i = 1}^{N}{\hat{{{{{{{{\mathcal{S}}}}}}}}}}_{i}$ as our estimator for the value of our scoring function ${{{{{{{\mathcal{S}}}}}}}}$, where *N* ~ 18 × 5 × 6 million is the total number of experimental rounds.

The Azuma–Hoeffding inequality implies that the probability *p* that dense coding or, equivalently, a two bit communication model will yield a value of ${{{{{{{\mathcal{S}}}}}}}}$ greater or equal to the observed value is bounded by

17 $$p\left(\frac{1}{N}\mathop{\sum }\limits_{i = 1}^{N}{\hat{{{{{{{{\mathcal{S}}}}}}}}}}_{i}\ge {{{{{{{{\mathcal{S}}}}}}}}}_{2{{{{{{{\rm{bits}}}}}}}}}+\mu \right)\le \exp \left(\frac{2N{\mu }^{2}}{{(c+T)}^{2}}\right) , $$

where *μ* = 0.379 is the observed violation of the two bit bound, *T* = 9 is the classical 2-bit bound on $-{{{{{{{\mathcal{S}}}}}}}}$ and $c\equiv {\max }_{xy}{c}_{xy}/p(x , y)$. One finds that this probability is vanishingly small. The analogous procedure applies to the data analysis based on the measured value of ${{{{{{{\mathcal{T}}}}}}}}$.

## Supplementary information

Supplementary Information

## Data Availability

Source data is provided as supplementary to this paper. Any additional data related to the findings of this paper is available from the corresponding author upon request.

## Code availability

The code used for the purposes of analysing data is also available from the corresponding author upon request.
