# Supplementary material for: Entanglement-assisted quantum communication with simple measurements
Source: Nat Commun. 2022 Dec 22;13:7878. doi: 10.1038/s41467-022-33922-5 (PMC9780301; doi:10.1038/s41467-022-33922-5)
Supplement: Supplementary file 1 — Supplementary Information [file 41467_2022_33922_MOESM1_ESM.pdf]

Entanglement-assisted quantum communication with simple measurements

Amélie Piveteau,[1] Jef Pauwels,[2] Emil Håkansson,[1,3] Sadiq Muhammad,[1,4] Mohamed Bourennane,[1] and Armin Tavakoli[5,6]

[1]*Department of Physics, Stockholm University, S-10691 Stockholm, Sweden*
[2]*Laboratoire d'Information Quantique, CP 225, Université libre de Bruxelles (ULB), Av. F. D. Roosevelt 50, 1050 Bruxelles, Belgium*
[3]*Hitachi Energy Research, Forskargränd 7, 72219 Västerås, Sweden*
[4]*Department of Applied Physics, Royal Institute of Technology (KTH), Stockholm 106 91, Sweden.*
[5]*Institute for Quantum Optics and Quantum Information - IQOQI Vienna, Austrian Academy of Sciences, Boltzmanngasse 3, 1090 Vienna, Austria*
[6]*Institute for Atomic and Subatomic Physics, Vienna University of Technology, 1020 Vienna, Austria*
(Dated: September 22, 2022)

Here we provide additional details on the experimental setup including details about the parties' unitary operations and how they are implemented, experimental error estimation and two-fold Hong-Ou-Mandel dip visibility.

SUPPLEMENTARY NOTE 1: RANDOM ACCESS CODE - $\mathcal{R}$

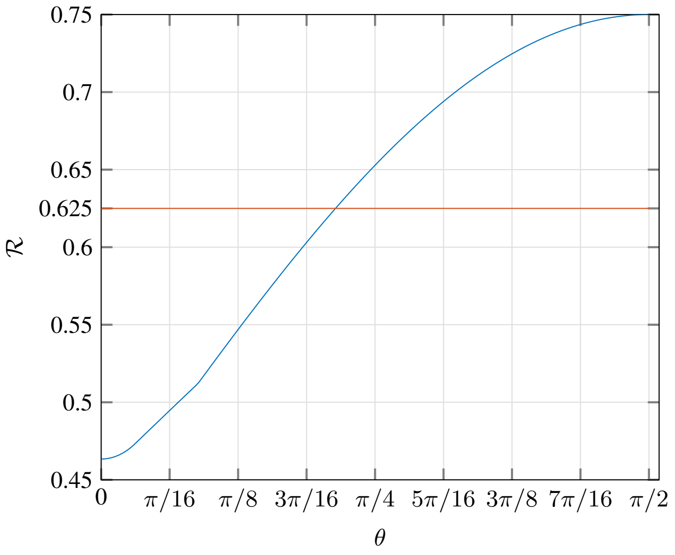

Supplementary Figure 1: Numerically obtained success rate in the Random Access Code versus the degree of entanglement in the shared state. The red horizontal line represents the best success rate based on 2 bits of communication. The success rate decreases as the entanglement weakens, because this reduces the dense coding capability of the channel. Above $\theta \gtrsim 0.6720$ the dense coding capability is still large enough to allow a quantum strategy to beat the success rate of 2 bit protocols.

Consider a Random Access Code based on multi-valued inputs [1]. The sender has a total of 16 inputs, expressed as two pieces of data $x_1, x_2 \in \{1, 2, 3, 4\}$. The receiver randomly selects the data of interest, either choosing $y = 1$ for x_1 or $y = 2$ for x_2. The aim is for the sender to encode (x_1, x_2) into a message such that the receiver can recover x_y with high average probability. The average success rate is given by $\mathcal{R} = \frac{1}{32}\sum_{x_1,x_2,y} p(b = x_y|x_1, x_2, y)$.

In the main text, we gave an explicit optimal strategy that achieves $\mathcal{R} = \frac{3}{4}$ in which the two measurements are mutually unbiased bases of maximally entangled states. It is interesting to note that in this quantum protocol, all winning probabilities are equal, i.e. $\forall(x_1, x_2, y)$ we have $p(b = x_y|x_1, x_2, y) = \frac{3}{4}$. Hence, the worst-case probability of recovering the data of interest is equal to the average probability $\mathcal{R}$.

If the EPR state is subject to isotropic noise, i.e. the effective state is $v|\phi^+\rangle\langle\phi^+| + \frac{1-v}{4}\mathbb{1}$, then the optimal strategy returns an advantage over protocols based on two bits of communication whenever $v > 3/4$. This is found immediately from solving $\frac{3}{4}v+(1-v)\frac{1}{4} = \frac{5}{8}$. The reason is that with probability v one plays the optimal strategy, achieving $\mathcal{R} = \frac{3}{4}$, while with probability $1 - v$ one randomly guesses the output, achieving $\mathcal{R} = \frac{1}{4}$, which has to equate to the optimal success rate based on 2 bits, which is $\mathcal{R} = \frac{5}{8}$ [2].

The use of a maximally entangled qubit pair is essential in order to convey two bits with a single qubit message. To further elucidate the relationship between information capabilities and correlations created, we instead assign a pure partially entangled state $|\psi_\theta\rangle = \cos\frac{\theta}{2}|00\rangle + \sin\frac{\theta}{2}|11\rangle$, for $\theta \in [0, \frac{\pi}{2}]$. One expects the ability to convey information to grow with θ, from sending one bit when $\theta = 0$ to sending two bits when $\theta = \frac{\pi}{2}$. A natural way to quantify the information capability of such a partially entangled state is to consider the success rate with which the state can be used in dense coding. In other words, the largest probability of recovering a four-valued message x by applying a unitary transformation U_x to one share of the state and then extracting the information via a quantum measurement $\{M_b\}$. We therefore write

$$\mathcal{D}(\psi_\theta) = \max \frac{1}{4}\sum_{x=1}^{4} p(b = x|x, \psi_\theta) = \max_{\{U_x\},\{M_b\}} \frac{1}{4}\sum_{x=1}^{4} \mathrm{tr}\left[(U_x \otimes \mathbb{1})\, |\psi_\theta\rangle\langle\psi_\theta|\, \left(U_x^\dagger \otimes \mathbb{1}\right) M_x\right]. \tag{1}$$

Following dense coding, we consider that the extraction measurement is a Bell basis measurement up to a local unitary, i.e. $M_b = |B_b\rangle\langle B_b|$ with $|B_b\rangle = V \otimes \mathbb{1}|\phi_b\rangle$, where the four states $|\phi_b\rangle$ are the Bell states $|\phi^+\rangle, |\phi^-\rangle, |\psi^+\rangle$ and $|\psi^-\rangle$. This simplifies the dense coding ability to

$$\mathcal{D}(\psi_\theta) = \max_{\{W_x\}} \frac{1}{4}\sum_{x=1}^{4} |\langle\phi_x|\,(W_x \otimes \mathbb{1})\,|\psi_\theta\rangle|^2, \tag{2}$$

where $W_x = V^\dagger U_x$. One can then see that the optimal unitaries are the same as in dense coding, namely $W_1 = \mathbb{1}$, $W_2 = \sigma_Z$, $W_3 = \sigma_X$ and $W_4 = \sigma_Y$. This gives $\mathcal{D}(\psi_\theta) = \frac{1+\sin\theta}{2}$. As expected, we see that the dense coding ability increases from 50% ($\theta = 0$) to 100% ($\theta = \frac{\pi}{2}$). We consider the critical degree of entanglement, i.e. the smallest value of θ, such that we can leverage a qubit message and a shared state $|\psi_\theta\rangle$ to beat the limit of a 2 bit strategy in the Random Access Code. We have numerically investigated this and the results are shown in Supplementary Figure 1. As expected, we find that the success rate decreases as the dense coding capability decreases. Nevertheless, we are able to outperform the limit $\mathcal{R} = \frac{5}{8}$ whenever $\theta \gtrsim 0.6720$. This corresponds to $\mathcal{D} \approx 81\%$.Thus, quantum resources of a significantly smaller capacity still outperform two bits of communication in the Random Access Code.

SUPPLEMENTARY NOTE 2: UNITARY OPERATIONS IN THE EXPERIMENT CORRESPONDING TO FIGURE OF MERIT $\mathcal{S}$

Depending on her setting x, the sender applies the unitary transformations U_x^{S}, given by

$$\begin{gathered} U_1^{\mathrm{S}} = \mathbb{1}, \quad U_2^{\mathrm{S}} = \frac{-\sigma_Z\sqrt{3} - \sigma_X}{2}, \quad U_3^{\mathrm{S}} = \frac{\sigma_X\sqrt{3} - \sigma_Z}{2}, \\ U_4^{\mathrm{S}} = \frac{\mathbb{1} - i\sigma_Y\sqrt{3}}{2}, \quad U_5^{\mathrm{S}} = \frac{\mathbb{1} + i\sigma_Y\sqrt{3}}{2}. \end{gathered} \tag{3}$$

The receiver, in turn depending on his setting y, applies the unitary transformation U_y^{R}, given by

$$\begin{gathered} U_1^{\mathrm{R}} = \mathbb{1}, \quad U_2^{\mathrm{R}} = \frac{\nu_+\mathbb{1} + i\nu_-\sigma_Y}{2\sqrt{2}}, \quad U_3^{\mathrm{R}} = \frac{\nu_+\mathbb{1} - i\nu_-\sigma_Y}{2\sqrt{2}}, \\ U_4^{\mathrm{R}} = U_2^{\mathrm{S}}, \quad U_5^{\mathrm{R}} = U_3^{\mathrm{S}}, \quad U_6^{\mathrm{R}} = \frac{\mathbb{1} - i\sigma_Y}{\sqrt{2}}, \end{gathered} \tag{4}$$

where $\nu_\pm = \sqrt{3} \pm 1$. These unitary operations are realised in the experiment by rotating a half wave plate with rotation angle θ combined with a phase shift ϕ,

$$U_z^{\mathrm{K}} = \begin{pmatrix} \cos(2\theta_{\mathrm{K}}^z) & \sin(2\theta_{\mathrm{K}}^z) \\ e^{i\phi_{\mathrm{K}}^z}\sin(2\theta_{\mathrm{K}}^z) & -e^{i\phi_{\mathrm{K}}^z}\cos(2\theta_{\mathrm{K}}^z) \end{pmatrix}, \tag{5}$$

where $z = x$ for $\mathrm{K} = \mathrm{S}$ and $z = y$ for $\mathrm{K} = \mathrm{R}$.

The rotation angles θ_{K}^z and phases ϕ_{K}^z corresponding to each setting for the sender and receiver are listed in Supplementary Table I and Supplementary Table II respectively.

SUPPLEMENTARY NOTE 3: EXPERIMENTAL RESULTS FOR $\mathcal{S}$

Supplementary Table III lists our experimental results alongside the theoretical probabilities for each combination of settings (x, y) associated to a non-zero payoff in the communication task ($c_{xy} \neq 0$). We also list the estimated errors, discussed below.

SUPPLEMENTARY NOTE 4: UNITARY OPERATIONS IN THE EXPERIMENT CORRESPONDING TO FIGURE OF MERIT $\mathcal{T}$

Depending on her setting x_1, x_2, x_3, the sender applies the unitary transformations U_x, given by:

$$U_x = (-1)^{x_1} \begin{pmatrix} -\alpha_{x_1}\mu_{x_2x_3} & (-1)^{x_2+x_3}\alpha_{\bar{x}_1}\mu_{x_2x_3} \\ (-1)^{x_2+x_3}\sqrt{2}\alpha_{\bar{x}_1} & \sqrt{2}\alpha_{x_1}, \end{pmatrix}, \tag{6}$$

where $\mu_{x_2x_3} = (-1)^{x_2} + i(-1)^{x_3}$, $\alpha_s = \frac{1}{2}\sqrt{1 + (-1)^s\sqrt{2/3}}$ and the bar-sign denotes bit-flip. These unitary operations are realised in the experiment by two half wave plates and two quarter wave plates combined with a phase shift. The receiver makes the following measurements:

$$E_1 = \sigma_Z \otimes \sigma_Z\,, \quad E_2 = \frac{1}{2}\sigma_Y \otimes (\sqrt{3}\sigma_Y + \sigma_Z)\,, \quad E_3 = \frac{1}{2}\sigma_X \otimes (\sqrt{3}\sigma_Y - \sigma_Z)\,. \tag{7}$$

These measurements are realised in the experiment by a half wave plate and a quarter wave plate on the photon receiving the unitary and with a half wave plate with two quater on the other photon. The rotation angles and phases corresponding to each setting for the sender and receiver are listed in Supplementary Table IV and Supplementary Table V respectively.Wave plates are named in the order of arrival for the photon, as shown in the figure in the main text.

SUPPLEMENTARY NOTE 5: EXPERIMENTAL RESULTS FOR $\mathcal{T}$

Supplementary Table VI lists our experimental results for each combination of unitary rotation $U_{x_1x_2x_3}$ and measurent E_y. We also list the estimated errors, as for the other experiment.

SUPPLEMENTARY NOTE 6: ERROR ESTIMATION

Following [3] we consider error originating from the measurement side only. To reduce experimental errors in the measurements, we used computer controlled high precision motorised rotation stages to set the orientation of wave-plates with repeatability precision $0.02°$ for the first experiment and $0.025°$ for the second experiment. The use of different settings (x, y) induces a systematic error, which we estimate using Monte Carlo simulation. We assume that the wave-plates setting error is normally distributed with a standard deviation of $0.02°$ for the first experiment and $0.025°$ for the second experiment. This together with the Poissonian error in photon counting statistics comprise the final error reported here. Due to inefficiency in the single photon detectors, the photons are detected randomly and their counting is Poissonian. To decrease Poissonian counting error, we have chosen a measurement time of two hours for every setting and collected about 18 Million events. To guarantee that both parties receive single qubits, we worked at a low rate ($\approx$ 2500 pairs per sec) to suppress higher order coincidence to almost 0.9 per sec.

SUPPLEMENTARY NOTE 7: TWO-FOLD HONG-OU-MANDEL DIP VISIBILITY

Bell state measurements are implemented through two-photon interference, using PBS and HWP plates set at 22.5°. The photons are detected by Si avalanche photodiodes and the coincidences are registered with an eight channels multifold coincidence counting unit. This Bell analyser consists of coherent interference at a polarisation beam splitter. To obtain indistinguishability of the photons, due to their arrival times, we adjusted the path length of one of the photons by using a delay line [4]. In Supplementary Figure 2, the coincidences between the detectors versus the delay path is shown.The zero delay corresponds to a maximal overlap (maximum indistinguishability). The interfering photons will bunch (they will exit only in one output arm of the PBS) causing the coincidence to vanish. The obtained visibility of the two-fold Hong-Ou-Mandel dip is 0.961 ± 0.002.

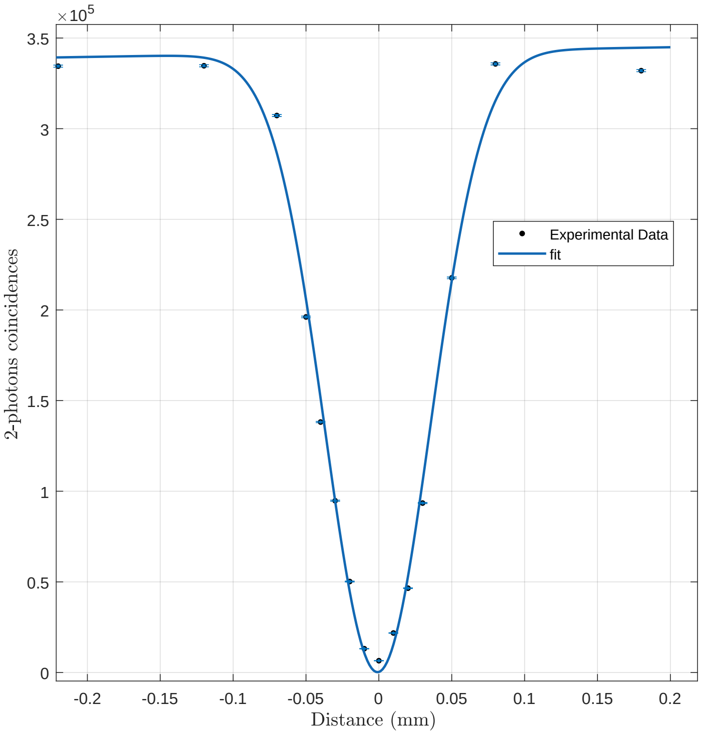

Supplementary Figure 2: Two-fold Hong-Ou-Mandel dip. The plot displays the two-fold photon counting coincidence versus the delay (the path difference between the two arms). The error bars indicate the Poissonian photon counting error statistics. The data is fitted with Gaussian function.

x	ϕ_{S}^{x}	θ_{S}^{x}
1	π	0
2	0	15
3	0	-30
4	π	-30
5	π	30

Supplementary Table I: Half wave plate rotation angles θ_{S}^{x} and phase shifts ϕ_{S}^{x} applied by the sender for different settings x.

y	ϕ_{R}^{y}	θ_{R}^{y}
1	π	0
2	π	7.5
3	π	-7.5
4	0	15
5	0	-30
6	π	-22.5

Supplementary Table II: Half wave plate rotation angles θ_{R}^{y} and phase shifts ϕ_{R}^{y} applied by the receiver for different settings y.

c_{xy}	$p(b\|x,y)$ Theory	$p(b\|x,y)$ Experiment	Errors
c_{11}	1	0.9725	0.0003
c_{21}	0	0.0250	0.002
c_{31}	0	0.0100	0.003
c_{12}	$(2+\sqrt{3})/4$	0.9087	0.0006
c_{42}	$(2-\sqrt{3})/4$	0.0610	0.001
c_{13}	$(2+\sqrt{3})/4$	0.9198	0.0006
c_{53}	$(2-\sqrt{3})/4$	0.0793	0.0009
c_{24}	1	0.9688	0.0003
c_{34}	0	0.0020	0.005
c_{44}	0	0.0050	0.004
c_{54}	0	0.0120	0.002
c_{35}	1	0.9828	0.0003
c_{45}	0	0.0240	0.002
c_{55}	0	0.0070	0.003
c_{46}	$(2+\sqrt{3})/4$	0.9068	0.0006
c_{56}	$(2-\sqrt{3})/4$	0.0560	0.001

Supplementary Table III: Experimental results for $\mathcal{S}$.

$U_{x_1x_2x_3}$	phase	H_1	Q_1	H_2	Q_2
U_{000}	π	-8.816	45	33.75	45
U_{001}	0	-8.816	45	-78.75	45
U_{010}	0	-8.816	45	-33.75	45
U_{011}	π	-8.816	45	78.75	45
U_{100}	π	53.816	45	33.75	45
U_{101}	0	53.816	45	-78.75	45
U_{110}	0	53.816	45	-33.75	45
U_{111}	π	53.816	45	78.75	45

Supplementary Table IV: Half and quarter wave plate rotation angles and phase shifts applied by the sender for different settings $U_{x_1x_2x_3}$.

y	Mode1 H_1	Mode1 Q_1	Mode2 Q_1	Mode2 H_1	Mode2 Q_2
1	0	0	0	0	0
2	0	-45	0	15	0
3	22.5	0	0	30	0

Supplementary Table V: Half and quarter wave plate rotation angles applied by the receiver on each arms for different settings y.

$U_{x_1x_2x_3}$	Measurement	$p(b = x_y\|x, y)$ Theory	$p(b = x_y\|x, y)$ Experiment	error
U_{000}	E_1	0.9082	0.9139	0.01
U_{000}	E_2	0.9082	0.864	0.01
U_{000}	E_3	0.9082	0.9062	0.01
U_{001}	E_1	0.9082	0.9535	0.01
U_{001}	E_2	0.9082	0.8968	0.01
U_{001}	E_3	0.9082	0.8532	0.01
U_{010}	E_1	0.9082	0.9814	0.01
U_{010}	E_2	0.9082	0.8458	0.01
U_{010}	E_3	0.9082	0.8808	0.01
U_{011}	E_1	0.9082	0.9087	0.01
U_{011}	E_2	0.9082	0.8753	0.01
U_{011}	E_3	0.9082	0.9081	0.01
U_{100}	E_1	0.9082	0.8979	0.01
U_{100}	E_2	0.9082	0.9057	0.01
U_{100}	E_3	0.9082	0.9044	0.01
U_{101}	E_1	0.9082	0.8787	0.01
U_{101}	E_2	0.9082	0.8746	0.01
U_{101}	E_3	0.9082	0.9356	0.01
U_{110}	E_1	0.9082	0.8247	0.01
U_{110}	E_2	0.9082	0.9136	0.01
U_{110}	E_3	0.9082	0.9266	0.01
U_{111}	E_1	0.9082	0.9113	0.01
U_{111}	E_2	0.9082	0.905	0.01
U_{111}	E_3	0.9082	0.9044	0.01
$\mathcal{T} = \frac{1}{24}\sum_{x,y} p(b = x_y\|x, y)$		0.9082	0.8988	0.003

Supplementary Table VI: Experimental results for $\mathcal{T}$.

SUPPLEMENTARY REFERENCES

[1] A. Tavakoli, A. Hameedi, B. Marques, and M. Bourennane, Phys. Rev. Lett. **114**, 170502 (2015).
[2] A. Ambainis, D. Kravchenko, and A. Rai, Optimal Classical Random Access Codes Using Single d-level Systems (2015), arXiv:1510.03045v1, 1510.03045.
[3] H. Anwer, S. Muhammad, W. Cherifi, N. Miklin, A. Tavakoli, and M. Bourennane, Phys. Rev. Lett. **125**, 080403 (2020).
[4] C. K. Hong, Z. Y. Ou, and L. Mandel, Phys. Rev. Lett. **59**, 2044 (1987).
